# Gamification in medical education

**DOI:** 10.3402/meo.v20.29536

**Published:** 2015-09-29

**Authors:** Maroof Ahmed, Yusuf Sherwani, Osama Al-Jibury, Muhammad Najim, Riham Rabee, Muhammad Ashraf

**Affiliations:** Faculty of Medicine, Imperial College London, London, UK

Technology is increasingly being used in medical education to supplement the delivery of learning resources. Gamification is defined as ‘the use of game design elements in non-game contexts’ (1). Despite being a relatively young field, gamification is making a big impact across a variety of industries and has piqued the interest of the academic community, with education being one of the first industries to introduce games as a complement to learning. However, the gamification of medical education has struggled to gain traction.

A systematic literature review conducted by Hamari et al. (2) has shown that gamification has proven effective in many fields, most notably education. Studies in education and learning contexts illustrate how the gamification of learning outcomes has been positive by increasing motivation and engagement in the learning tasks, as well as enjoyment over time. However, the studies also highlighted some negatives that require attention, such as the effects of increased competition, task evaluation difficulties, and design features.

There are many examples of the successful implementation of gamification in a variety of fields. For example, American Airlines offering rewards in their frequent-flyer programme in 1982, or more recently Foursquare, a location-based social network, using gamification successfully to reward users for ‘checking-in’ (3). Foldit, a game released by the University of Washington, has demonstrated a successful use case of gamification in the context of education. The public was asked to play a protein-folding exercise to elucidate structures of various proteins; in 10 days, the players were able to unlock the crystal structure of a monomeric retroviral protease that causes AIDS in rhesus monkeys, a feat with which scientists had struggled for 15 years (4).

There are also examples of successful use cases in the university setting, such as the gamification of the *Social Media Innovation* course at the Fox School of Business at Temple University. Steven L. Johnson gamified the learning process with a mission-based narrative where students leveled up after a certain number of accomplishments, such as achievements or badges (5). Another example is the *Just Press Play* initiative at the Rochester Institute of Technology. Game elements and a narrative outline were used to create an educational game that promoted academic success (6).

In the last decade, there has been increasing interest in medical educational strategies, with the development of new concepts such as problem-based learning, student-centered learning, and integrated teaching. Many theories exist regarding the next move for medical education. Given the success of gamification in other educational settings, the implementation of game elements in medical education could provide an innovative solution. Further research is needed to evaluate the effectiveness of gamification in medical education.

*Maroof Ahmed* Faculty of Medicine, Imperial College London, London, UK Email: maroof.ahmed11@imperial.ac.uk *Yusuf Sherwani* Faculty of Medicine, Imperial College London, London, UK *Osama Al-Jibury* Faculty of Medicine, Imperial College London, London, UK *Muhammad Najim* Faculty of Medicine, Imperial College London, London, UK *Riham Rabee* Faculty of Medicine, Imperial College London, London, UK *Muhammad Ashraf* Faculty of Medicine, Imperial College London, London, UK
